# A chromosome-level genome assembly of the parasitoid wasp *Eretmocerus hayati*

**DOI:** 10.1038/s41597-023-02450-2

**Published:** 2023-09-06

**Authors:** Yu-Wei Zhong, Yun-Yun Fan, Zhang-Qi Zuo, Run-Guo Shu, Yin-Quan Liu, Jun-Bo Luan, Fei Li, Shu-Sheng Liu

**Affiliations:** 1 Key Laboratory of Biology of Crop Pathogens and Insects of Zhejiang Province, Institute of Insect Sciences, Zhejiang University, 310058 Hangzhou, China; 2 College of Plant Protection, Shenyang Agricultural University, 110866 Shenyang, China

**Keywords:** Genome, Sequencing

## Abstract

Hymenoptera is an order accounting for a large proportion of species in Insecta, among which Chalcidoidea contains many parasitoid species of biocontrol significance. Currently, some species genomes in Chalcidoidea have been assembled, but the chromosome-level genomes of Aphelinidae are not yet available. Using Illumina, PacBio HiFi and Hi-C technologies, we assembled a genome assembly of *Eretmocerus hayati* (Aphelinidae, Hymenoptera), a worldwide biocontrol agent of whiteflies, at the chromosome level. The assembled genome size is 692.1 Mb with a contig N50 of 7.96 Mb. After Hi-C scaffolding, the contigs was assembled onto four chromosomes with a mapping rate of > 98%. The scaffold N50 length is 192.5 Mb, and Benchmarking Universal Single-Copy Orthologues (BUSCO) value is 95.9%. The genome contains 370.8 Mb repeat sequences and total of 24471 protein coding genes. P450 gene families were identified and analyzed. In conclusion, our chromosome-level genome assembly provides valuable support for future research on the evolution of parasitoid wasps and the interaction between hosts and parasitoid wasps.

## Background & Summary

Hymenoptera is a mega-diverse insect order with >169,000 described species, and a large proportion of species in this order are parasitoid wasps, such as the species of Chalcidoidea, that play an important role in the biological control of insect pests. Derived from the same ancestor that lived in the Permian or Triassic, parasitoid wasps have evolved numerous branches with unique characteristics to adapt to different living environments^1^.

Within Hymenoptera, over 30 species genomes in Chalcidoidea have been assembled hitherto, and some of them were assembled at the chromosome level, including *Aphelinus atriplicis* (Aphelinidae), *Aphelinus certus* (Aphelinidae)^2^, *Muscidifurax raptorellus* (Pteromalidae)^3^, *Nasonia vitripennis* (Pteromalidae)^4,5^, *Pteromalus puparum* (Pteromalidae)^6^, *Theocolax elegans* (Pteromalidae)^7^, *Valisia javana* (Agaonidae)^8^, *Anastatus japonicus* (Eupelmidae), *Anastatus fulloi* (Eupelmidae)^9^, *Megastigmus duclouxiana* (Megastigmidae) and *Megastigmus sabinae* (Megastigmidae)^10^. Among these, the chromosome-level assemblies of *A. atriplicis* and *A. certus* were generated according to a genetic map of hybrids between each other^2^.

*Eretmocerus hayati* (Aphelinidae, Hymenoptera) is an obligate parasitoid of the global prominent pest whitefly *Bemisia tabaci* (Aleyrodidae, Hemiptera)^11–13^. The release of this parasitoid wasp can effectively control the outbreak of whitefly^14–16^. The parasitoid wasps of the genus *Eretmocerus* have unique parasitic habits. With a curved tip on their ovipositor, they can lay eggs between the host nymphs and plant leaves instead of directly into host body^17^. The hatched first instar larva breaks through the abdomen of host, but still lives outside the host body, and does not burrow into the host body to complete the subsequent development until the host has developed to the last nymphal stage^18,19^. The first and second instar larvae are wrapped by the capsule structure produced by the host, and will not fully contact the host tissue until the third instar^18^.

Here, we report a chromosome-level genome assembly of *E. hayati* using combined Illumina, PacBio and Hi-C sequencing technology. The assembly is 692.1 Mb in length with a scaffold N50 of 192.5 Mb and BUSCO completeness 97.4%. 53.58% repeat sequences and 24471 protein coding sequences were identified. We also identified 74 P450 genes and discovered the expression patterns at different development stages and sex. This assembly provides a valuable resource for evolutionary and host-parasitoid interaction studies in parasitoid wasps as well as biological control application of *E. hayati*.

## Methods

### Sampling and genome sequencing

**Fig. 1:**
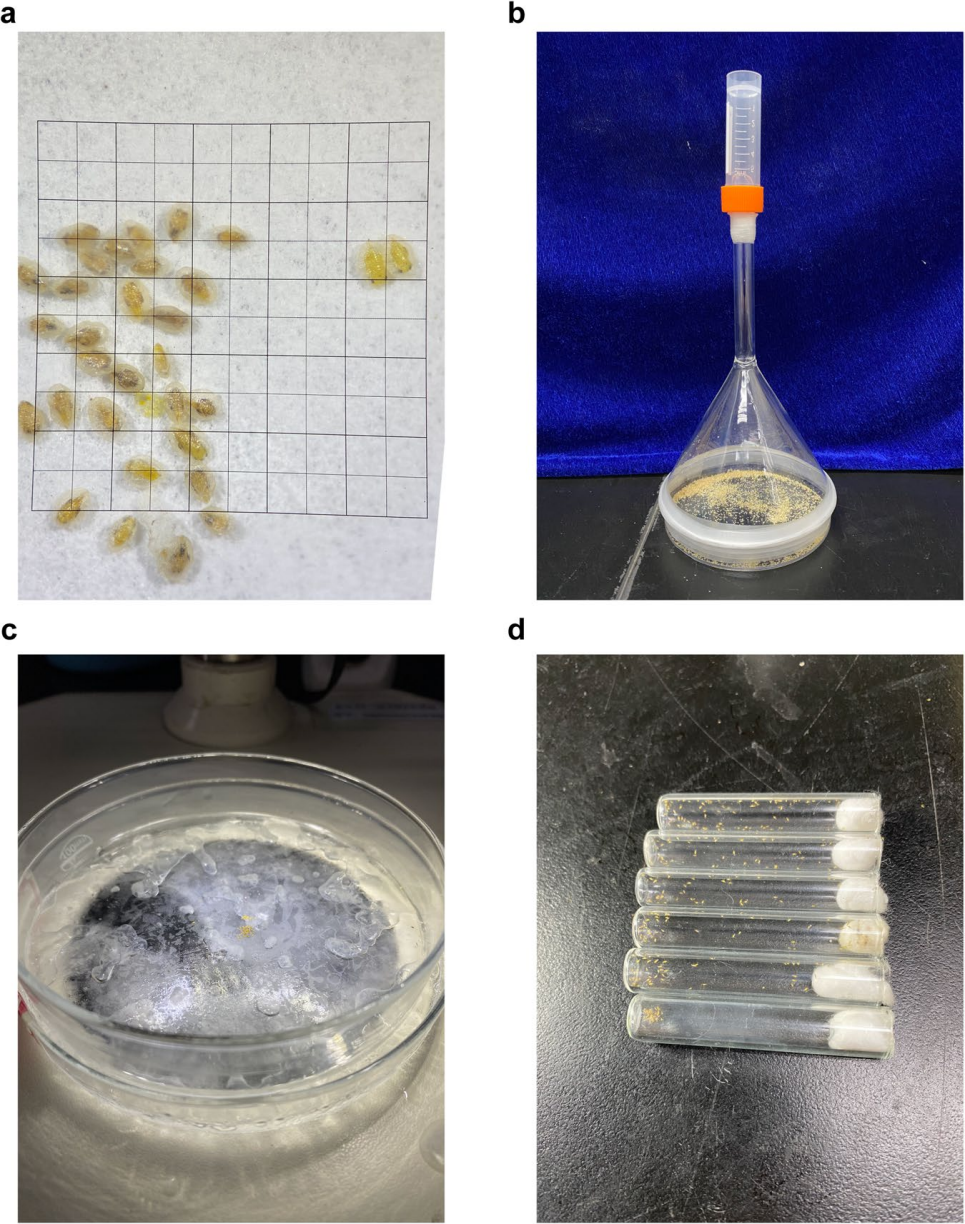
Device and process for collecting adult male wasps. (**a**) Pupae of *E. hayati*. Male pupae are on the left and two female pupae on the right. The sides of the small square are 1.0 mm in length. (**b**) Device for collecting newly emerged parasitoid wasps. Pupae were collected and placed in the petri dish, and the newly emerged parasitoid wasps would move upward into the sampling tube. (**c**) Device for distinguishing the sex of parasitoid wasps and collecting male wasps. Newly emerged wasps were placed in a petri dish sitting on ice and were anesthetized for examination and collection. (**d**) Tubes for resuscitating parasitoid wasps. Anesthetized male wasps were collected and placed in these tubes for resuscitation.

A strain of *E. hayati* was initially imported by the Chinese Academy of Agricultural Sciences in 2008 and had been reared on *Gossypium hirsutum* plants carrying whitefly nymphs in our laboratory (26 ± 1°C, 14 L:10D, 70 ± 10%RH) over seven years. To collect the newly emerged parasitoid wasps, the pupae of *E. hayati* were first collected in a petri dish, then a triangle funnel of appropriate size was inversely placed on the petri dish with a 1.5 ml plastic sampling tube on the outlet of the funnel neck (Fig. 1a,b). We anesthetized the emerged parasitoid wasps on ice and collected approximately 3000 newly emerged males for DNA extraction using QIAamp DNA Mini Kit (QIAGEN) (Fig. 1c,d). After extraction, the DNA purity, concentration and integrity were detected using NanoDrop 2000&8000, Qubit fluorescence photometer and Agilent 4200 Bioanalyzer, respectively.

A short-read sequencing library with an insert size of 350 bp was generated using Universal DNA Fragmentase kit V2.0 (Annoroad) and Universal DNA Library Prep Kit V2.0 (Annoroad) with 0.5 ug DNA as input material. Hiseq x ten platform was used to sequence the library and generate 47.65 Gb paired-end clean reads after removing adapters and low-quality reads.

For long-read library, the SMRT bell express template prep kit 2.0 (Pacific Biosciences) was used to construct a SMRT bell library using 8 ug sheared DNA. After size-selection, primer annealing and binding the SMRT bell templates to polymerases, the library was sequenced on the Pacific Bioscience Sequel II platform in Annoroad Gene Technology company (TianJin). Single SMRT cells were processed, generating 481.17 Gb of subreads data.

### Estimation of genome size

Flow cytometry was performed as described below to estimate the genome size. The heads of *E. hayati* were cut off with a dissecting needle after parasitoid wasps were anesthetized on ice. Next, we collected 300 heads as one sample in a 1.5 mL centrifuge tube and ground them with an abrasive rod and 200 μL Galbraith’s nuclear dissociation solution^20^. Then the fully ground tissue solution was filtered with a 38 μm pore size nylon mesh to obtain the nuclear suspension. Then, the cells were centrifuged at 1000 r/min for 5 min at 4 °C.

Next the precipitated nuclei were resuspended with 400 μL 1 × PBS buffer by lightly scratching at the bottom of the tube. Finally, we added PI solution (final concentration was 50 μg/mL) and RNase A solution (final concentration was 20 μg/mL) to the nuclei, and stained the samples at 4 °C in dark for 5–20 min. Heads of *Drosophila melanogaster* (genome size is 176.4 Mb) were collected as a reference to calculate the size of the genome.

**Fig. 2:**
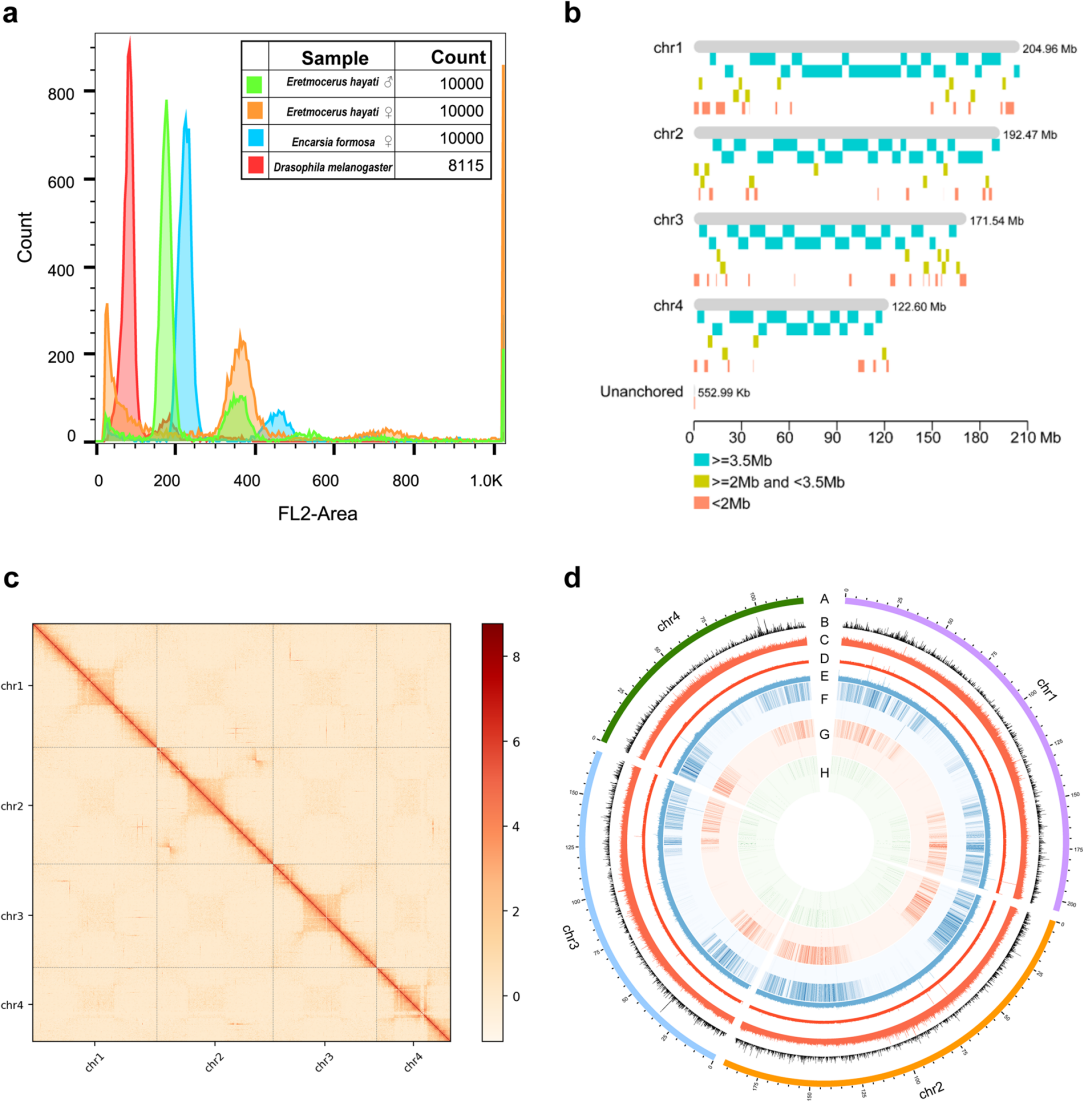
Genome assembly of *Eretmocerus hayati*. (**a**) Flow cytometry results of 4 samples. The ordinate is the number of nuclei, and the abscissa is the fluorescence of the nucleus. *Drosophila melanogaster* was used as reference sample. (**b**) Contig distribution on genome chromosomes. Grey bars represent different lengths of the corresponding chromosome. Rectangles of other colors represent contigs of different lengths loading on the chromosomes. (**c**) Genome-wide all by all Hi-C interaction heatmap of *E. hayati* (4 chromosomes, resolution 100 kb). The intensity of chromosomal interactions is shown on the right shading gradient. Intrachromosomal interactions (red blocks in the diagonal) are much stronger than interchromosomal interactions (light yellow blocks). (**d**) Chromosome-level genome assembly results information circle plot (window size 50 kb). A: chromosome information, B: gene density, C: GC content, D: second-generation sequencing depth, E: third-generation sequencing depth, F: heterozygous SNP distribution (outer) and homozygous SNP distribution (inner), G: heterozygous Indel distribution (outer) and homozygous Indel distribution (inner), H: single copy (outer) and multiple copy (inner) BUSCO genes distribution.

Flow cytometry results show that the DNA content in the nucleus of the female *E. hayati* was four times that of the female *Drosophila melanogaster*, indicating that the estimated genome size was about 756.227 Mb (Fig. 2a, Table 1).

**Table 1:** Summary of flow cytometry results.

Sample	Peak Ch	The ratio to the reference sample	Estimated genome size (Mb)
*Drasophila melanogaster*	87	1	176.400
*Eretmocerus hayati* female	373	4.287	756.227
*Eretmocerus hayati* male	177	2.034	717.595
*Encarsia formosa* female	236	2.713	478.573

We also used k-mer distribution analysis^21^ to estimate the genome size and characteristics of *E. hayati*. For k-mer analyze, 47.65 Gb clean reads from the short-read library were used with K-mer size of 21. The genome size of *E. hayati* was estimated to be 694.3 Mb by 21 k-mer analysis, with a heterozygosity of about 0.01% and a repeat sequence ratio of 40.94% (Table 2).

**Table 2:** The K-mer analysis result of the *Eretmocerus hayati* genome.

Sample	*Eretmocerus hayati*
K-mer	21
K-mer number	36,796,517,662
K-mer depth	53
Estimated genome size (bp)	694,273,918
Repeat (%)	40.94
Heterozygous ratio (%)	0.01

### Genome assembly

We used HiFiasm v0.15.1 to preliminarily assemble the *E. hayati* genome, which could resolve near-identical repeats and segmental duplications to generate better haplotype assemblies^22^. The HiFiasm outputs a primary assembly after performing all-versus-all read overlap alignment and correcting sequencing errors.

Purge Haplotigs software was used to complete genome de-redundancy after initial assembly and error corrected, and the redundant heterozygous contigs were identified and removed according to reads depth distribution and sequence similarity^23^.

The hybrid set with 47.65 Gb Illumina short read sequencing (67-fold coverage) and 481.17 Gb PacBio sequencing reads (693-fold coverage) (Table 3) was used to obtain a *de novo* genome assembly for *E. hayati* with total length of 763.42 Mb and contig N50 length of 7.96 Mb (Table 4).

**Table 3:** Summary of sequencing data for genome assembly.

Library	Raw data (bp)	Clean data (bp)	Estimated coverage (X)
Illumina	47,647,246,800	46,669,306,500	67.2
PacBio HiFi	482,156,146,021	481,172,257,121	693.3
Hi-C	70,004,377,500	68,096,753,747	98.1

^*^Assumed genome size to be 694 Mb.

**Table 4:** Summary of assembly results of *de novo* and after Hi-C scaffolding for *Eretmocerus hayati*.

	Length (bp)		Number	
	*De novo*	Hi-C scaffolds	*De novo*	Hi-C scaffolds
Total	763,424,385	692,130,109	244	17
Max length	32,738,843	204,958,428	—	—
Number(> = 2 kb)	—	—	244	17
N50	7,961,343	192,470,813	30	2
N60	5,980,150	171,544,537	41	3
N70	4,440,234	171,544,537	56	3
N80	3,144,237	171,544,537	76	3
N90	2,093,294	122,604,546	105	4

### Hi-C scaffolding

To obtain the genome at the chromosomal level, Hi-C technology was applied^24^. We commissioned Annoroad (TianJin) to construct a Hi-C sequencing library using more than 1500 adult male wasps. Firstly, DNA containing biotin was captured under the adsorption of avidin magnetic beads and terminal repair of DNA fragments was performed. Then poly A was added to the end of the DNA fragment, the joint was connected, the number of PCR amplification cycles was evaluated, and the final purification library was built. The library was sequenced on the Illumina platform and generated 70 Gb sequencing data (98-fold coverage), which was used to construct the genome at the chromosome level.

We used bowtie 2 (v2.2.3)^25^ to map the paired-end reads to the preliminary assembly. Then, HiC-Pro (v2.7.8)^26^ was used to detect the ligation site of unmapped reads, which were mainly composed of the chimeric regions spanning across the ligation junction. The 5’ fractions of unmapped reads were aligned back onto the genome. Next, in order to generate a high quality alignment file, we discarded the low mapping quality, multiple hitting reads and singletons according to the mapping results. To obtain the chromosome-scale scaffolds from the primary contig-level assembly, we used 3d-DNA^27^ and LACHESIS^28^ to treat the valid interaction pairs and generate the interaction matrices. According to the results of the karyotype study of Chalcidoidea, the subfamily Aphelininae harbored parasitoids with n = 4–5^29^. We consulted the literature and the output results of 3D-DNA to assess the number of chromosomes, which appears to be four. To ascertain the precision of the outcomes, we built the interaction graph with Juicer^30^ and performed manual visual error correction using JuiceBox (v2.13.07) (https://github.com/aidenlab/Juicebox). After that, we cut the pseudochromosomes into equal 100 kb length bins and used Hic-Pro to constructe the heatmap showing the significant linkage clusters on diagonal.

The 244 contigs were divided, anchored, sorted, oriented, and merged into 4 chromosomes using LACHESIS and corrected by JuiceBox (Fig. 2b), with 13 sequences unanchored. The chromosomal heat map showed good collinearity on the diagonal (Fig. 2c), which confirms the high quality of scaffolding. The final genome assembly was 692.1 Mb with a scaffold N50 of 192.5 Mb (Table 4, Fig. 2d).

### Genome completeness and quality assessment

We used three methods to assess the completeness and quality of the assembly. First, the genome sequence was split into 1000 bp fragments, and then these fragments were BLASTN against the NCBI nucleotide database (NT library). Second, we used BWA (v0.7.17)^31^ and Minimap2 (v2.24)^32^ to compare the second and third generation sequencing data with the genome, and calculated the comparison rate and sequence coverage. Third, BUSCO (v5.3.2)^33^ was used to estimate the gene space of chromosome-level genome assembly by searching the 1367 BUSCO genes in insecta_odb10.

The best five hits of BLASTN again NCBI nt database were from *Nasonia*, *Gossypium*, *Eretmocerus*, *Ceratosolen* and *Copidosoma* (Table 5). 96.09% of the Illumina short reads and 100% of the PacBio HiFi long reads were mapped to the genome assembly successfully. Moreover, we compared the insect_odb10 database using BUSCO. The assessment showed 95.9% of BUSCO genes were successfully detected, of which 94.6% were single copy and 1.3% were duplicated (Table 6). The results of these evaluations indicate that the genome assembly has a high level of completeness and accuracy.

**Table 5:** Blast search of interrupted contig sequences in NCBI NT database.

Genus	Blast number	Total blast number	Hit percentage (%)
*Nasonia*	2,076	12,976	16.01
*Gossypium*	1,898	12,976	14.64
*Eretmocerus*	733	12,976	5.65
*Ceratosolen*	590	12,976	4.55
*Copidosoma*	508	12,976	3.92

**Table 6:** BUSCO assessment of the *Eretmocerus hayati* genome assembly.

Category	Number of BUSCOs
Complete BUSCOs (C)	1,311
Complete and single‐copy BUSCOs (S)	1,293
Complete and duplicated BUSCOs (D)	18
Fragmented BUSCOs (F)	8
Missing BUSCOs (M)	48
C: 95.9% [S: 94.6%, D: 1.3%], F: 0.6%, M: 3.5%	1,367

*using insect_odb10 as reference database. Chromosome-level assembly as input genome.

In addition, the alignment results of second generation sequencing data were used to identify SNP and INDELS using GATK (v4.4.0.0)^34^ with hard filtering parameters “–filter-expression “QD < 2.0 || FS > 60.0 || SOR > 3.0 || MQRankSum < −12.5 || ReadPosRankSum < −8.0””. VCFtools (0.1.16)^35^ was used to calculate the density of SNP and INDELS on the assembly, and window size was 50,000 bp.

### RNA sequencing and analysis

A total of 17 RNA-seq libraries were constructed, including four different development stages and various tissues of *E. hayati*. We extracted total RNA from egg (<post-deposition, 2000 individuals, single sample), 1^st^ instar larva (1-2 d after hatching and 5–6 d after the eggs were first deposited, still attached to the leaf, 1000 individuals, three replicates), later instar larva (7–8 d after hatching and 10–11 d after the eggs were first deposited, mostly 3^rd^-4^th^ instar within the body of whitefly nymphs and sampled by dissecting whitefly nymphs, 100 individuals, three replicates), female adult (<12 h post emergence and without exposure to male wasps, 150 individuals, three replicates), male adult (<12 h post emergence and without exposure to female wasps, 150 individuals, three replicates), female head (anaesthetized and dissected on ice with the method described above, 600 individuals, single sample), and male head (anaesthetized and dissected on ice with the method described above, 600 individuals, single sample). Total RNA was used for library preparations, which were sequenced on an Illumina Novaseq platform. All 150 bp paired-end raw reads were processed and a total of 98.5 Gb (approximately 14 Gb per sample) clean reads data were obtained. Then the clean reads were used to annotate the genome.

### Repeat sequence annotation

We identified repeat sequences and transposable elements (TEs) using the methods of *de novo* assembly and homologous prediction. First, we used RepeatModeler (v2.0.3) (https://github.com/Dfam-consortium/RepeatModeler) to predict the repeat sequence with default parameters. Then RepBase database^36^ and RepeatMasker (v4.1.0) (https://github.com/rmhubley/RepeatMasker) were used to annotate the sequence homologous.

The results showed that 370.8 Mb are repeat sequences, accounting for 53.58% of the *E. hayati* genome. Among these repeat sequences, most (32.39%) are unclassified elements, followed by 8% of long terminal repeats (LTRs), 7.27% of DNA elements, 5.63% of long interspersed nuclear elements (LINEs) and only 0.29% of short interspersed nuclear elements (SINEs) (Table 7).

**Table 7:** Summary of repeat elements annotation for *Eretmocerus hayati*.

Category	Number of elements	Length occupied (bp)	Percentage of genome (%)
SINEs	7,875	1,990,318	0.29
LINEs	45,677	38,969,627	5.63
LTR elements	60,693	55,396,603	8.00
DNA elements	124,695	50,344,896	7.27
Unclassified	555,939	224,151,700	32.39
Total		370,853,144	53.58

### Gene prediction and function assignment

We annotated protein coding genes in the *E. hayati* genome using a pipeline that combines *de novo* prediction, homology searching and transcriptome evidence^37^. The *de novo* prediction was implemented with BRAKER (v2)^38^. For details, we randomly selected 10000 intact *E. hayati* genes from the transcriptomes to generate prediction parameters using AUGUSTUS (v3.1)^39^, GeneMark-ET Suite (v4.21)^40^, and SNAP (v2006-07-28)^41^. After that, the newly trained parameters were applied to the repeat sequence masked genome to predict genes following the BRAKER pipeline. For homology searching, the protein sequences of *Nasonia vitripennis*^5^ and *Encarsia Formosa* were TBLASTN (v2.8.1) (E-value < 1e-5) against the *E. hayati* genome^42^. Then, the gene models were defined by aligning the genome sequences to the matched protein using GeneWise (v2.4.1)^43^. For transcriptome evidence, we aligned the RNA-seq reads to the *E. hayati* genome by Hisat2 (v2.2.1)^44^ to identify gene information such as candidate exon regions, donor, and acceptor sites. StringTie^45^ was used to assemble the alignments into transcripts. Finally, the above three lines of gene prediction evidence were integrated with EVidenceModeler (EVM)^46^ to generate a consensus gene set. Specifically, the weight for different methods were “4”, “5” and “10” for *de novo* prediction, homology searching, and transcriptome evidence, respectively.

We used several convention methods to annotate the function of predicted protein coding genes. First, gene ontology (GO) annotation was generated by using both PANNZER2^47^ web server and BLAST2GO (v5)^48^ with default parameters. Second, online service of BLASTKOALA (v2.2)^49^ was used to map the protein sequences of *E. hayati* to Kyoto Encyclopedia of Genes and Genomes (KEGG) pathways with database parameter “family_eukaryotes”. InterProScan (v5.57-90.0)^50^ was used to obtain motifs and domains information of protein sequences by searching the default available database (e.g. CDD, Gene3D, PANTHER, Pfam and SMART). To obtain function prediction of protein sequences, we combined the results of eggNOG-mapper (v2)^51^ and blastp against SwissProt and Trembl database (E-value < 1e-5).

A total of 24,471 protein coding genes were annotated following the pipeline combined with above-mentioned three methods. In total, 7,803 genes could be assigned with GO terms and 7,159 genes with KEGG ID. In addition, 16808 genes have BLASTN hits in NCBI non-redundant database, 11,556 genes contain Pfam domains (Table 8). Finally, a total number of 23974 genes were annotated by at least one database. Gene density was generated by TBtools (v1.120)^52^.

**Table 8:** Summary of gene annotation results for *Eretmocerus hayati*.

Category	Number of genes	Percentage of all predict genes (%)
NCBI (nr)	16,808	68.69
GO	7,803	31.89
KEGG	7,159	29.26
Pfam	11,556	47.22
Total	23,974	97.97

*Total: genes annotated by at least one database.

### Cytochrome P450 genes

P450 genes are key genes closely related to insect stress resistance and environmental adaptation. *E. hayati* shows many unique and interesting features in association with host. For example, its eggs and first instar larvae survive *in vitro*, and the subsequent stages survive inside the host body. We hope to infer the mechanism of its unique parasitic habits through the identification and expression pattern analysis of P450 genes. To identify the P450 genes in the *E. hayati* genome, we first downloaded P450 protein sequences from UniPortKB, GeneBank and FlyBase to build a reference database. All predicted protein sequences of *E. hayati* were used as query to BLASTP against the P450 reference sequences. The Blast hits were retained for further domain analysis using InterProScan. Finally, genes with P450 conservative domain pf00047 were retained and classified according to the BLASTP analysis results.

To reconstruct the phylogeny tree of P450 genes from *E. hayati*, *E. Formosa* and *N. vitripennis*, we used MAFFT (v7.471)^53^ with the parameter “-auto” to align P450 sequences. The alignment was filtered by TRIMAL (v1.4.rev22)^54^ with the parameter “-automated1”,and IQ-tree (v2.1.2)^55^ with parameters “-m MFP -bb 1000” was used to construct the maximum likelihood (ML) phylogenetic tree with the best model (JTT + F + R8) estimated by ModelFinder^56^. RSEM (v1.3.3)^57^ was used to map the transcriptome clean data to the P450 sequences to estimate expression level of P450 genes in various tissues.

**Fig. 3:**
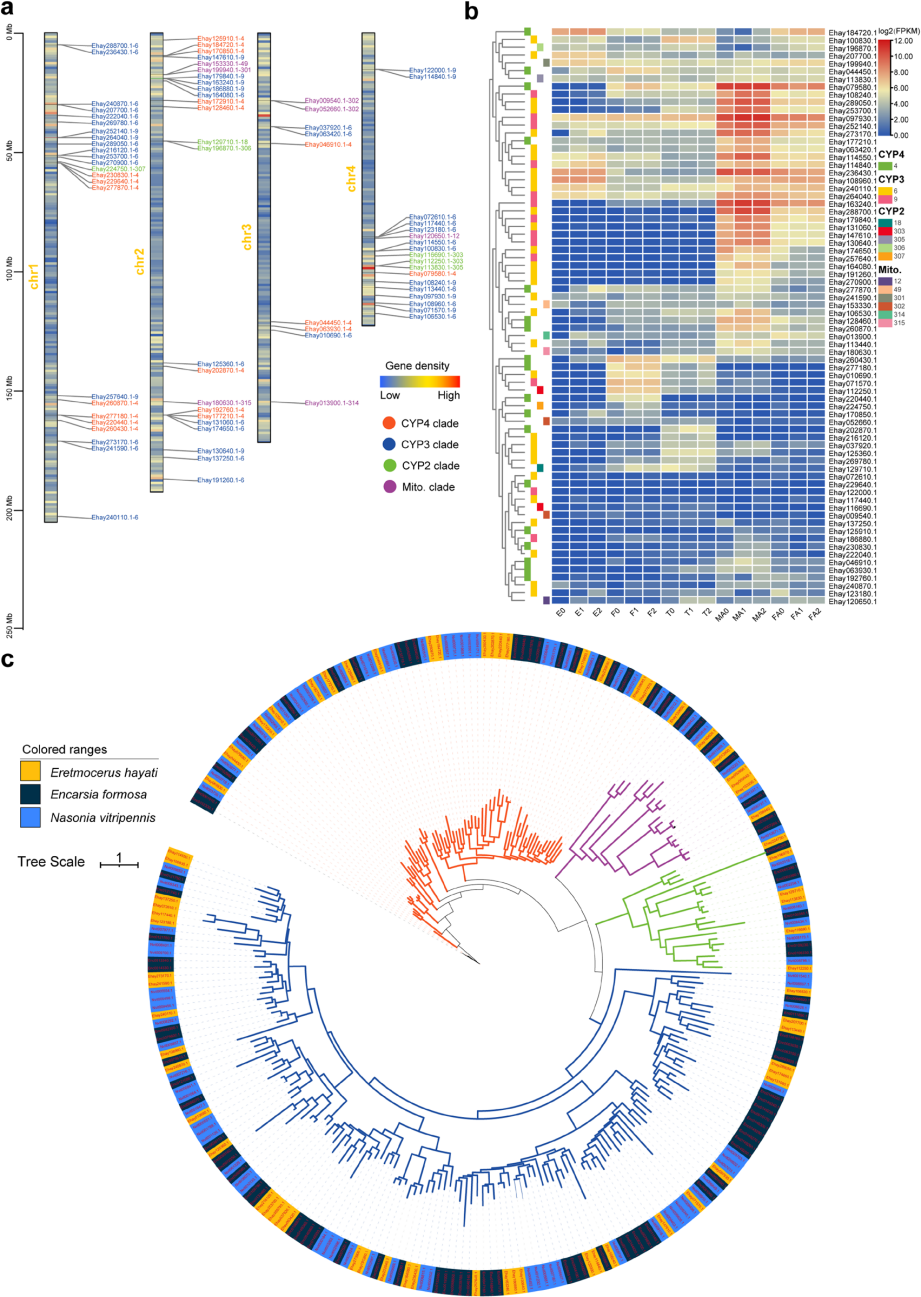
Chromosomal location, classification and expression of P450 genes. (**a**) The location of the P450 genes on chromosomes. The density of the chromosomal genes is displayed by stripes of different colors. The superfamily type of the P450 gene is represented by the text color of the gene name, orange represents CYP4, blue represents CYP3, green represents CYP2, and purple represents Mito. (**b**) The maximum-likelihood phylogenetic tree of P450 genes derived from *E. hayati*, *E. formosa* and *N. vitripenni*s using IQ-TREE software. The branch colors indicate different P450 superfamilies, consistent with the above description. (**c**) FPKM values (log2 transformed) of P450 genes in different tissues. Gene superfamilies are marked on the left using colored squares and tissue types are marked below (E: eggs; F: 1^st^ instar larvae; T: later instar larvae; FA: female adults; MA: male adults; numbers represent biological replicates).

In total, we annotated 74 P450 genes in *E. hayati*, among which 6 genes belong to CYP2 clan, 42 belong to CYP3 clan, 19 belong to CYP4 clan, and 7 belong to Mito clan (Table 9). Chromosome mapping results show that the P450 genes are distributed on all four chromosomes, with more on chromosome 1, 2 and 4, and fewer on chromosome 3. There are six gene clusters containing three or more P450 genes on four chromosomes (Fig. 3a). The results of the phylogenetic tree show that the P450 genes from *E. hayati*, *E. formosa* and *N. vitripennis* expanded in the CYP3 and CYP4 branches (Fig. 3b).

**Table 9:** P450 genes identified in *Eretmocerus hayati*.

CYP clan	CYP family	Gene ID			
2	305	Ehay113830.1			
	303	Ehay116690.1	Ehay112250.1		
	18	Ehay129710.1			
	306	Ehay196870.1			
	307	Ehay224750.1			
3	6	Ehay010690.1	Ehay037920.1	Ehay063420.1	Ehay174650.1
		Ehay072610.1	Ehay100830.1	Ehay106530.1	Ehay269780.1
		Ehay108960.1	Ehay113440.1	Ehay114550.1	Ehay288700.1
		Ehay117440.1	Ehay123180.1	Ehay125360.1	Ehay253700.1
		Ehay131060.1	Ehay191260.1	Ehay207700.1	Ehay273170.1
		Ehay216120.1	Ehay222040.1	Ehay236430.1	Ehay241590.1
		Ehay240110.1	Ehay164080.1	Ehay289050.1	Ehay270900.1
		Ehay240870.1			
	9	Ehay097930.1	Ehay108240.1	Ehay114840.1	Ehay071570.1
		Ehay122000.1	Ehay130640.1	Ehay147610.1	Ehay264040.1
		Ehay163240.1	Ehay179840.1	Ehay186880.1	Ehay257640.1
		Ehay252140.1			
4	4	Ehay044450.1	Ehay229640.1	Ehay172910.1	Ehay170850.1
		Ehay046910.1	Ehay230830.1	Ehay177210.1	Ehay220440.1
		Ehay063930.1	Ehay260430.1	Ehay184720.1	Ehay277870.1
		Ehay079580.1	Ehay260870.1	Ehay192760.1	Ehay128460.1
		Ehay125910.1	Ehay277180.1	Ehay202870.1	
Mito.	12	Ehay120650.1			
	49	Ehay153330.1			
	301	Ehay199940.1			
	302	Ehay009540.1	Ehay052660.1		
	314	Ehay013900.1			
	315	Ehay180630.1

The expression patterns of P450 genes differ significantly among various tissues. Some CYP3 clan genes are expressed in all stages, but the expression levels are significantly higher in adult male wasps than in other stages (e.g. Ehay097930.1, Ehay252140.1, and Ehay264040.1). Some CYP3 clan genes are expressed only in the adult stage and are expressed at higher levels in male than in females (e.g. Ehay163240.1, Ehay288700.1, and Ehay270900.1). Some P450 genes show relatively higher expression levels in the 1^st^ instar larvae (e.g. Ehay260430.1 and Ehay170850.1), and others show higher expression levels in later instar larvae (e.g. Ehay202870.1 and Ehay269780.1) (Fig. 3c).

P450 genes are highly related to responses to external stimuli and stress, and whether the different expression levels of P450 in different stages of *E. hayati* are related to the parasitic behavior of the wasp *in vivo* and *in vitro* deserves further study.

## Data Records

Illumina, PacBio and Hi-C data for *E. hayati* genome sequencing have been deposited in the NCBI Sequence Read Archive with accession number SRR24094107, SRR24099399 and SRR24094118 under BioProject accession number PRJNA951478^58^.

Illumina transcriptome data for egg (SRR24094058, SRR24094059, SRR24094060), 1^st^ instar larva (SRR24094061, SRR24094062, SRR24094063), later instar larva (SRR24094064, SRR24094050, SRR24094051), female adult (SRR24094052, SRR24094053, SRR24094054), male adult (SRR24094055, SRR24094056, SRR24094057), female head (SRR24094065) and male head (SRR24094066) are available under Bioproject PRJNA951478^58^.

This Whole Genome Shotgun project has been deposited at GenBank under the accession JARUXH000000000^59^. The version described in this paper is version JARUXH010000000.

The annotation file is available in figshare^60,61^.

## Technical Validation

After extraction, the DNA purity, concentration and integrity were detected using NanoDrop 2000&8000, Qubit fluorescence photometer, and Agilent 4200 Bioanalyzer (Agilent Technologies, CA, USA), respectively. RNA integrity was assessed using the RNA Nano 6000 Assay Kit of the Bioanalyzer 2100 system (Agilent Technologies, CA, USA). High-quality DNA and RNA were used for sequencing.

## Data Availability

All software and scripts were executed according to user manual, and default parameters were applied if not mentioned in the Methods described above.
